# Radiation dosimetry and biodistribution in non-human primates of the sodium/iodide PET ligand [^18^F]-tetrafluoroborate

**DOI:** 10.1186/s13550-015-0148-5

**Published:** 2015-12-03

**Authors:** J. M. Marti-Climent, M. Collantes, M. Jauregui-Osoro, G. Quincoces, E. Prieto, I. Bilbao, M. Ecay, J. A. Richter, I. Peñuelas

**Affiliations:** Department of Nuclear Medicine, Clínica Universidad de Navarra, Avda. Pío XII, 36, 31008 Pamplona, Spain; Instituto de Investigación Sanitaria de Navarra (IdiSNA), Pamplona, Spain; Small Animal Imaging Research Unit, Center for Applied Medical Research (CIMA) - Clínica Universidad de Navarra, Pamplona, Spain; Division of Imaging Sciences, King’s College, London, UK; Fundación Centro Nacional de Investigaciones Cardiovasculares (CNIC) - CIBER de Enfermedades Respiratorias (CIBERES), Madrid, Spain

**Keywords:** PET, Tetrafluoroborate, Sodium/iodide symporter, Thyroid, Radionuclide imaging, Dosimetry, Non-human primate

## Abstract

**Background:**

[^18^F]-tetrafluoroborate is a PET radiotracer taken up by the sodium/iodide symporter (NIS). Albeit the in vivo behavior in rodents is similar to the ^99m^Tc-pertechnetate, no studies exist in primates or in humans. The aims of this study were to evaluate the biodistribution of [^18^F]-tetrafluoroborate in non-human primates with PET and to estimate the absorbed dose in organs.

**Methods:**

Whole-body PET imaging was done in a Siemens ECAT HR+ scanner in two male *Macaca fascicularis* monkeys. After an i.v. injection of 24.93 ± 0.05 MBq/kg of [^18^F]-tetrafluoroborate, prepared by isotopic exchange of sodium tetrafluoroborate with [^18^F]-fluoride under acidic conditions, eight sequential images from the head to the thigh (five beds) were collected for a total duration of 132 min. The whole-body emission scan was reconstructed applying attenuation and scatter corrections. After image reconstruction, three-dimensional volumes of interest (VOIs) were hand-drawn on the PET transaxial or coronal slices of the frame where the organ was most conspicuous. Time-activity curves for each VOI were obtained, and the organ residence times were calculated by integration of the time-activity curves. Human absorbed doses were estimated using the OLINDA/EXM software and the standard human model.

**Results:**

[^18^F]-tetrafluoroborate was able to discriminate clearly the thyroid gland with an excellent signal-to-noise ratio. Most of the radiotracers (residence time) are localised in the organs that express NIS (stomach wall, salivary glands, thyroid, olfactory mucosa), are involved in excretion (kidneys and bladder), or reflect the vascular phase (heart and lungs). Considering the OLINDA source organs, the critical organs were the stomach wall, thyroid and bladder wall, with absorbed doses lower than 0.078 mGy/MBq. The effective dose was 0.025 mSv/MBq.

**Conclusions:**

[^18^F]-tetrafluoroborate is a very useful radiotracer for PET thyroid imaging in primates, with a characteristic biodistribution in organs expressing NIS. It delivers an effective dose slightly higher than the dose produced by ^99m^Tc-pertechnetate but much lower than that produced by radioiodine in the form of ^131^INa, ^123^INa, or ^124^INa.

**Electronic supplementary material:**

The online version of this article (doi:10.1186/s13550-015-0148-5) contains supplementary material, which is available to authorized users.

## Background

The sodium/iodide symporter (NIS) is a plasma membrane glycoprotein that mediates the active transport of iodide in the thyroid and other NIS-expressing cells. NIS also mediates active iodide transport in other tissues, including salivary glands, gastric mucosa and lactating mammary glands. Due to its expression in only a small number of tissues, NIS provides the basis for the effective diagnostic and therapeutic management of thyroid cancer and its metastases with radioiodine [1–3]. More recently, tumor-selective expression of exogenous NIS has been proposed as a new strategy to deliver high absorbed doses selectively to tumors using radioiodine therapy for non-thyroidal cancers [4, 5]. Additionally, NIS has also been used as an imaging reporter gene [6].

Traditionally, imaging of NIS for diagnosis of disease and monitoring treatment for thyroid cancer and hyperthyroidism has been performed using the gamma emitters [^131^I]iodine and [^123^I]iodine. However, imaging of these radionuclides has limitations due to the reduced resolution and sensitivity of SPECT. Moreover, the relative long half-life of these radionuclides produces undesirable high absorbed doses. SPECT images with ^99m^Tc-pertechnetate are of high resolution, but PET technique offers better resolution and sensitivity than SPECT images and, moreover, allows a quantitative analysis. More recently, [^124^I]iodine, a positron emitter, has been applied in dose planning in radionuclide therapy [7, 8]. However, PET imaging of NIS with ^124^I has also some drawbacks. Iodine-124 has a complex decay scheme with a low abundance of positrons (23 %). It also emits high energy γ photons, some of which are very close to the 511 keV energy window or compete in abundance with the positron decay (*E*_max_ = 1.69 MeV, 10 %). These may interfere with annihilation photons leading to increased background noise and poor image quality. Moreover, the availability of iodine-124 is severely limited due to its complex production. Finally, its long half-life (4.18 days) results in an unjustifiably high absorbed dose to the patient.

In contrast, the ideal PET tracer for NIS would be based on ^18^F because of its good imaging characteristics: short half-life (110 min), high positron yield (97 %), low positron energy (*E*_max_ = 0.634 MeV) and ready availability by production on a medical cyclotron. In this context, the [^18^F]-tetrafluoroborate PET radiotracer has been synthesised by isotopic exchange with tetrafluoroborate ion (BF_4_^−^) under acidic conditions [9]. BF_4_^−^ is a substrate for NIS, as has been demonstrated by electrochemical studies and accumulation in the thyroid of rats [10, 11]. The usefulness of this new radiotracer to visualise thyroid and other organs expressing NIS has previously been described in preclinical PET/CT images in rodents [9].

Due to the broad applications of NIS in diagnostic and therapeutic strategies, a PET radiotracer for its imaging would be of great utility [12]. To promote the clinical application of this novel radioligand in humans, we studied the biodistribution and calculated the absorbed radiation exposure after intravenous administration of [^18^F]-tetrafluoroborate in cynomolgus monkeys (*Macaca fascicularis*). Primates were used in this preclinical investigation as their metabolism and physiology are closer to those of humans than those of rodents, and hence, extrapolation to humans is more reasonable.

## Methods

### Radiopharmaceutical production

The synthesis of [^18^F]-tetrafluoroborate was carried out using an automated labeling protocol developed as described earlier [9, 13]. Briefly, [^18^F]-fluoride trapped in a QMA cartridge was eluted with 1.5 mol/L HCl into a reactor that contained sodium tetrafluoroborate; the reaction mixture was heated to 120 °C for 10 min, cooled to 25 °C, passed through a silver ion-loaded cation exchange cartridge and two Sep-Pak Light Alumina N and sterile filtered. The product was sterile and pyrogen-free.

### Subjects/experimental animals

All animal experiments were performed in accordance with the European Council Directive 86/609/EEC and according to a protocol approved by the Ethical Committee for Animal Testing of the University of Navarra (ref: 026/11). Two male healthy cynomolgus monkeys (*M. fascicularis*, 4.47 and 7.06 kg) were used in this study. The animals fasted overnight with free access to water up to 2 h before the PET scan. Anaesthesia was initially induced by intramuscular injections of ketamine (10 mg/kg) and midazolam (1 mg/kg) to allow preparation and handling of the animals and maintained with a mixture of ketamine (5 mg/kg) and midazolam (0.5 mg/kg). Once in the PET facility, the animals were placed on the scanner bed in a prone position and safely attached to keep the position during the whole scan. The animals were covered with blankets to maintain body temperature. Two intravenous lines on both saphenous veins were inserted, one for the administration of the radiotracer and the other to obtain blood samples during the study. The heart rate, respiration and body temperature were monitored and kept in the normal range throughout the imaging sessions.

### Data acquisition

**Fig. 1:**
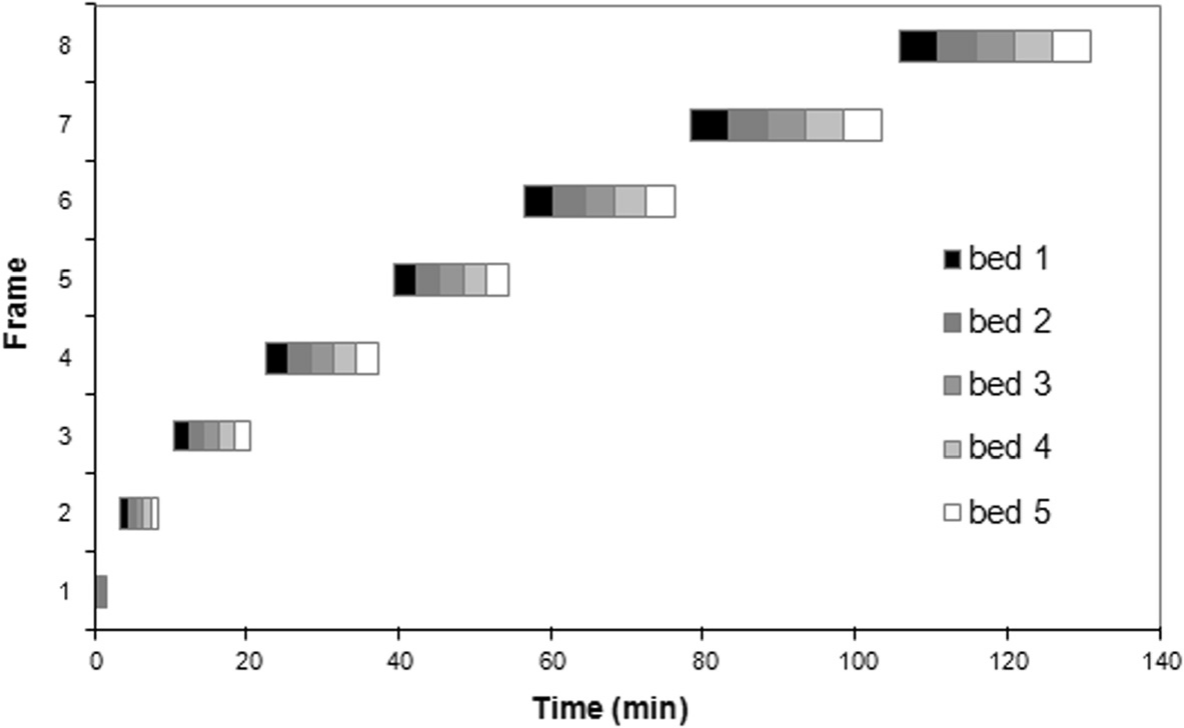
Scanning sequence for dynamic whole-body acquisition. Outline of the scanning sequences. Time required for repositioning and starting each frame was approximately 30 s

Imaging of each animal consisted of eight whole-body PET studies acquired successively using an ECAT EXACT HR+ scanner (Siemens/CTI, Knoxville, TN) in a static 2D mode. A scan from a single bed position comprises 63 slices with a 2.46-mm slice thickness, for covering an axial field of view of 15.5 cm [14]. The total axial field of view scanned for emission and transmission scans was 69 cm, encompassing the whole body of the animals. This was accomplished by acquiring five bed positions with an overlap of seven slices. Prior to tracer injection, a whole-body transmission scan was obtained using ^68^Ge rod sources for 5 min at each bed position. The attenuation correction factors were obtained after segmentation of the measured reconstructed attenuation map. Image acquisition started immediately after intravenous administration of 24.93 ± 0.05 MBq/kg [^18^F]-tetrafluoroborate. Each whole-body frame increased the duration over time for each position to compensate for ^18^F half-life. The time between successive bed positions was 12 s and between whole-body frames was about 30 s. The scanning sequence is presented in Table 1 and Fig. 1. The total duration of the emission scanning was 132 min, and the total duration of the transmission and emission scanning was approximately 160 min.

**Table 1:** Acquisition sequences for PET studies

Frame	Start time (min)	Time per bed (s)
1	0	15
2	3.5	60
3	10.5	120
4	22.5	180
5	39.5	180
6	56.5	240
7	78.5	300
8	106	300

Venous blood samples were collected at 0, 0.25, 4, 10, 21, 35, 50, 70, 115 and 130 min after [^18^F]-tetrafluoroborate administration. The blood samples were weighed and radioactivity measured in a NaI(Tl) well counter, calibrated with a ^68^Ge sample cross-calibrated with the germanium source used for the standard PET scanner calibration.

### PET reconstruction

The 2D whole-body emission scans were reconstructed using the ordered-subset expectation maximisation algorithm (two iterations and eight subsets) applying attenuation and scatter corrections (ECAT 7.2.2 software, Siemens/CTI). A Gaussian post reconstruction filter with a 4-mm FWHM kernel was applied. A brain mode and a zoom factor of 2 were also applied in the reconstruction. The five bed positions of each WB acquisition (128 × 128 × 63 matrix) were assembled in a volume with 128 × 128 × 269 voxels, with a 2.6 × 2.6 × 2.6 mm^3^ voxel size.

### Image analysis

Image analysis was performed using PMOD 3.0 software (PMOD Technologies Ltd, Zurich, Switzerland; www.pmod.com). The eight whole-body ECAT images were assembled to obtain a dynamic study file. Three-dimensional volumes of interest (VOIs) were contoured on the PET transaxial or coronal slices. After a careful visual inspection of the images, VOIs were drawn on the emission frame where the organs were most clearly seen, depending on organ uptake kinetics. Lung VOIs were drawn on the transmission image. The identifiable source organs analysed were as follows: lungs, brain, heart, kidneys, stomach, urinary bladder, thyroid, olfactory mucosa, esophagus, salivary glands (parotid, sublingual, submandibular) and whole body. The VOIs were projected onto each whole-body PET image and visually verified. Then, the total activity of each organ VOI and frame was determined.

In order to have a proper calibration of the scanner for monkey’s acquisition, the organ activity values were corrected for recovery relative to a large VOI that included the entire body for each frame, as previously described [15]. Thus, the activity value for each source organ at every time point was scaled to the measured recovery for the individual frames (decay corrected).

The activity in each organ VOI was decayed to the bed midtime reversing the ^18^F decay correction done by the ECAT reconstruction software to each bed within each frame. The organs extending through two bed positions in the assembled whole-body image were decayed to the midtime between the corresponding beds.

Time-activity curves (TAC) expressing the percentage of the injected dose (% ID) at the measured time (bed midtime) were obtained by dividing the VOI activity by the administered activity of [^18^F]-tetrafluoroborate.

### Residence time and absorbed dose calculations

For each organ, the TAC of the percentage of injected activity was plotted versus time. Data were fitted by trapezoidal approximations followed by exponential decay with the physical half-life from the last measured radioactivity concentration, assuming that the decline in radioactivity after this time point occurred by physical decay only, without any further biologic clearance, thus obtaining more conservative values than those obtained adjusting mathematical functions to the experimental values. The area under the curve is equivalent to the residence time.

The human absorbed dose in each organ and effective dose were estimated using the residence times and the model for a 70-kg adult in the OLINDA/EXM 1.1 software program (Vanderbilt University) [16]. It was assumed that the biodistribution in the monkey was the same as it would be in humans. Then, no scaling of the macaque biodistribution data was used to estimate human absorbed doses. No loss of urine or faecal matter was observed in these animals during the PET study. Thus, corrections to the absorbed dose calculations for radioactivity loss through these routes were not needed.

The residence times for the spleen and red marrow were calculated from the blood residence time. For the spleen, the residence time was calculated from the blood fraction of the spleen, which is 1.5 % of its volume [17]. For the red marrow, its residence time is taken from [18, 19]:

1 $$ {A}_{\mathrm{rm}}=0.19/\left(1\hbox{--} 0.39\right){A}_{\mathrm{Blood}} $$

The blood residence time was estimated using the TAC of the drawn samples, assuming that 5.4 % of the monkey’s mass was blood and subtracting the fractions assigned to the spleen and red marrow.

No bladder voiding was assumed in the dosimetry calculation, as the monkeys were not catheterised during the whole-body PET studies. We estimated the biologic half-life in the bladder by a fit to a single exponential increase-to-maximum fit to the decay corrected bladder TAC. It was considered that the [^18^F]-tetrafluoroborate clearance through the urinary pathway was complete unless the activity accumulated in the stomach at the end of the PET study. These parameters were used to estimate the residence time for [^18^F]-tetrafluoroborate in the bladder using the dynamic bladder model in the OLINDA/EXM software program, with the assumption of a 2.4-h voiding interval.

Absorbed dose estimates for organs not available within the phantom employed in OLINDA/EXM (parotid and submandibular glands) were performed using the unit-density sphere model incorporated in the software. Thus, the OLINDA/EXM-generated dose coefficients for ^18^F for a time-integrated activity coefficient (residence time) of 1 MBq-h/MBq were fitted against the sphere masses using a power-law model of the form:

2 $$ d=a\ {m}^b $$

where *d* is the dose coefficient, *a* and *b* are fitting parameters and *m* is the sphere mass [20]. Using these fitting parameters, we calculated the mean organ absorbed dose per unit administered activity for each part of the paired organ by multiplying the output of Eq. (2) by the corresponding residence time. The density and radionuclide distribution were assumed to be uniform in this method of calculation. For each part of the parotid and submandibular salivary glands, the individual mass used were 18 and 9.9 g, considering the mean volumes reported by Jentzen et al. [21] and a 1 g/cm^3^ density.

The residence times for all of the source organs were summed and subtracted from the theoretic total residence time value of *T*_1/2_/ln(2) to calculate the residence time of the remainder in the body, where *T*_1/2_ is the radioactive half-life of ^18^F (1.83 h).

## Results

### Radiochemistry

[^18^F]-tetrafluoroborate was prepared in greater than 95 % radiochemical purity as shown by radio–thin-layer chromatography and radio–high-performance liquid chromatography.

### Biodistribution and [^18^F]-tetrafluoroborate kinetics

**Fig. 2:**
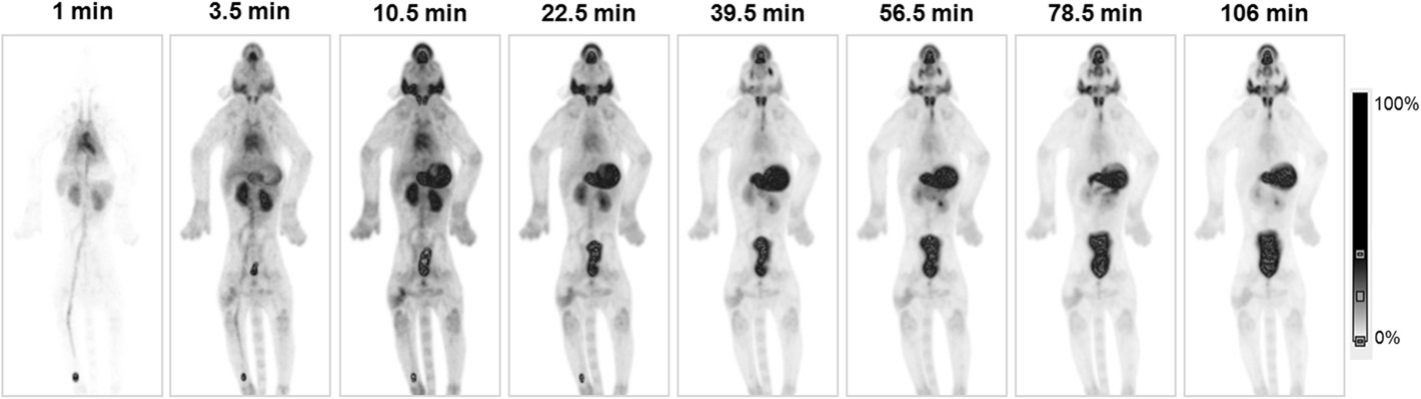
[^18^F]-tetrafluoroborate biodistribution in macaque. Maximum-intensity projection PET images of the [^18^F]-tetrafluoroborate radioactivity at different time points after intravenous injection. Time information corresponds to the mean frame time

Dynamic PET revealed the pattern and kinetics of [^18^F]-tetrafluoroborate distribution and clearance during the 132 min after a bolus intravenous administration. The whole-body images of [^18^F]-tetrafluoroborate biodistribution at different time intervals are shown in Fig. 2 and in a 3D video (Additional file 1). [^18^F]-tetrafluoroborate was able to discriminate clearly the thyroid gland with an excellent signal-to-noise ratio even at the first minutes of the study (frame 2, 3, 5 min). In addition, uptake was observed in the organs that also express NIS, like the stomach wall, salivary glands (mainly parotids) and olfactory mucosa. Some radioactivity was detected in the esophagus from minute 40 and hypothesised to originate from the swallowing of saliva. The stomach wall was the organ with the highest uptake; however, this uptake was not homogeneous, distinguishing two different areas. A slight activity was observed in the choroid plexus, but not in the testes. Finally, since [^18^F]-tetrafluoroborate is excreted via the urinary route, cumulative activity was observed in the urinary bladder.

**Fig. 3:**
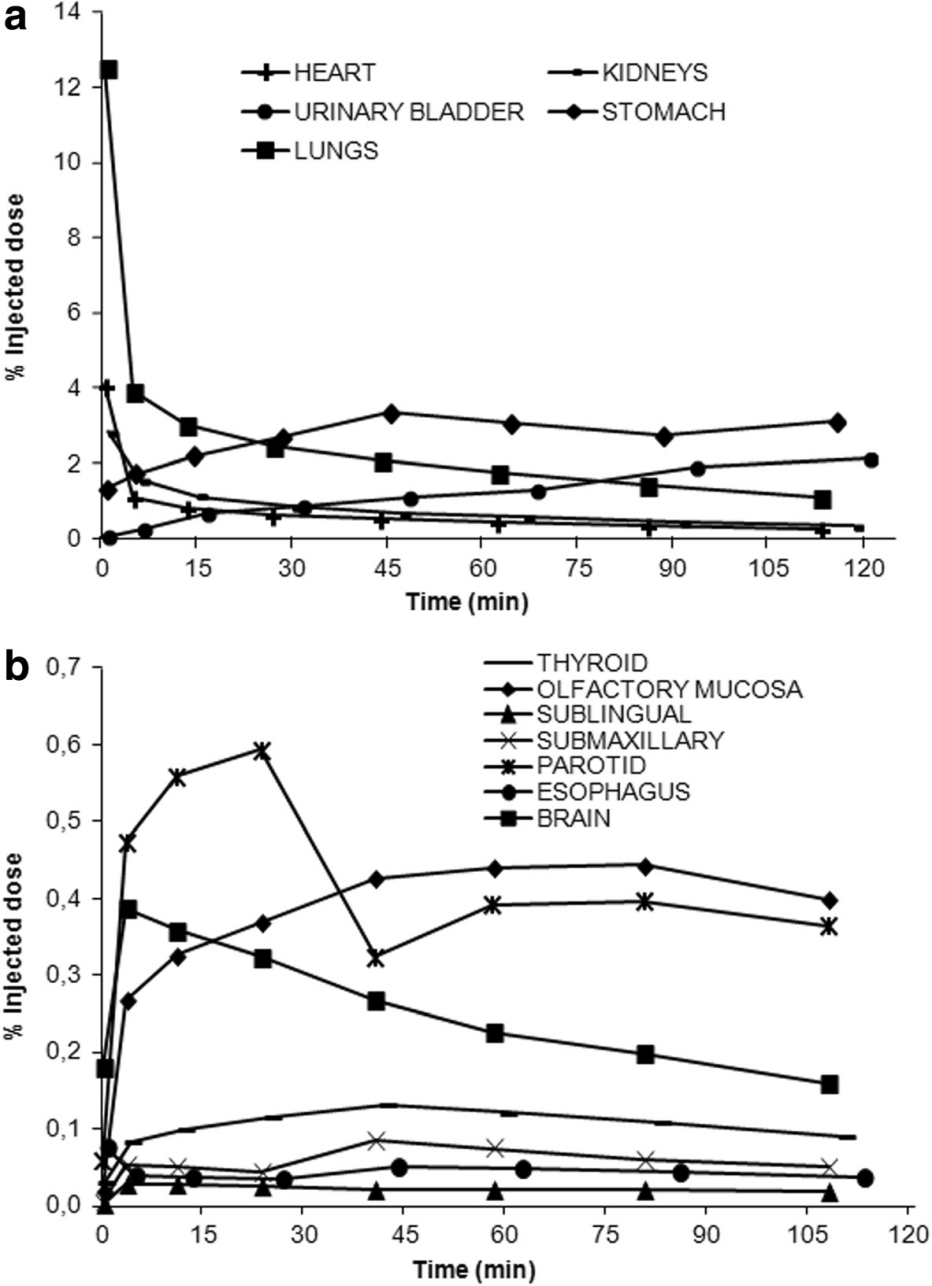
TACs for [^18^F]-tetrafluoroborate in organs. TACs for [^18^F]-tetrafluoroborate in the most prominent organs: lung, heart, kidney, stomach and urinary bladder (**a**) and thyroid, olfactory mucosa, esophagus, brain and salivary glands (parotid, sublingual, submandibular) (**b**). Plots **a** and **b** have different ranges of radioactivity concentrations. Curves correspond to a single animal. TACs from the other monkey showed a similar pattern

**Fig. 4:**
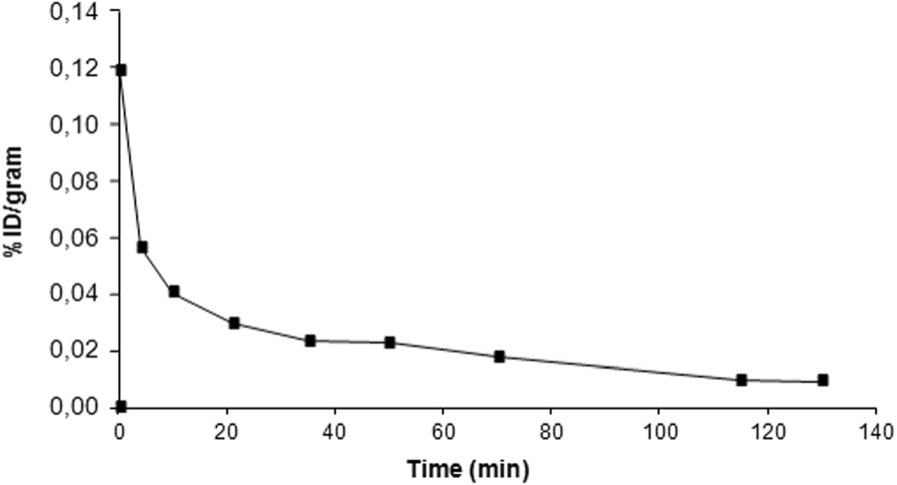
TACs for [^18^F]-tetrafluoroborate in blood. TACs for [^18^F]-tetrafluoroborate expressed as percentage of the injected dose (ID) per gram of blood. Curves correspond to a single animal

Individual source organ TACs for the percentage of the [^18^F]-tetrafluoroborate-injected activity are shown in Fig. 3, reflecting the visual analysis. Initial peaks of radioactivity in the heart and lungs are related to the vascular phase, and relative important accumulation trend was observed in the urinary bladder and stomach. Other organs expressing NIS also had accumulation, but in smaller amounts. In the case of thyroid, the uptake remained stable during the study. On the other hand, the total blood radioactivity concentration peaked within the first minute after the injection of [^18^F]-tetrafluoroborate and gradually decreased thereafter (Fig. 4). Both animals showed a similar biodistribution pattern.

### Absorbed dose estimate

Residence time of the selected organs in male cynomolgus macaques are shown in Table 2. The stomach, red marrow, urinary bladder and lungs had long residence times. The organs within the VOIs were clearly visible compared to the background but accounted for less than one quarter of the maximum theoretical residence time (2.635 h). Most of the injected tracer (~75 %) localised outside the VOIs modeled in the phantoms used by OLINDA/EXM, suggesting a low concentration of tracer distributed in the large compartments and organs and considered as “remainder” by OLINDA/EXM.

**Table 2:** Residence times of [^18^F]-tetrafluoroborate for the measured organs and remainder of the body

Organ	Residence time (h)	
	Mean	SD
Brain	1.21E−02	3.7E−03
Stomach	1.81E−01	5.3E−02
Heart contents	2.31E−02	3.9E−03
Kidneys	2.44E−02	8.4E−04
Lungs	6.79E−02	1.2E−02
Thyroid	4.68E−03	6.3E−06
Urinary bladder contents	7.66E−02	3.3E−02
Spleen	4.18E−03	7.0E−06
Red marrow	8.65E−02	1.4E−04
Remainder	2.14E+00	1.0E−01
Parotid gland	4.68E−03	2.5E−03
Submandibular gland	1.75E−03	1.1E−03

Results of the OLINDA/EXM 1.1 dose calculations for the PET studies of [^18^F]-tetrafluoroborate are given in Table 3. The data are presented as mean ± SD. The relative contribution of the source organs to the absorbed dose for a given tissue is determined by their respective *S* values. The stomach wall received the highest absorbed dose (0.079 mGy/MBq), making it the limiting organ. The other key organs with high absorbed dose were the urinary bladder wall (0.048 mGy/MBq), thyroid (0.042 mGy/MBq) and kidneys (0.022 mGy/MBq). The effective dose was 0.0247 mSv/MBq.

**Table 3:** Radiation-absorbed dose estimates for [^18^F]-tetrafluoroborate based on whole-body imaging in non-human primates

Target organ	Dose (mGy/MBq)	
	Mean	SD
Adrenals	1.44E−02	7.1E−05
Brain	4.54E−03	5.3E−04
Breasts	9.52E−03	2.8E−04
Gallbladder wall	1.46E−02	7.1E−05
LLI wall	1.42E−02	1.4E−04
Small intestine	1.43E−02	0.0E+00
Stomach wall	7.86E−02	1.9E−02
ULI wall	1.42E−02	0.0E+00
Heart wall	1.77E−02	7.1E−04
Kidneys	2.25E−02	3.5E−04
Liver	1.27E−02	2.8E−04
Lungs	1.78E−02	2.1E−03
Muscle	1.14E−02	2.1E−04
Ovaries	1.44E−02	0.0E+00
Pancreas	1.85E−02	1.1E−03
Red marrow	1.66E−02	1.4E−04
Osteogenic cells	2.01E−02	4.9E−04
Skin	8.68E−03	2.1E−04
Spleen	1.42E−02	7.8E−04
Testes	1.12E−02	7.1E−05
Thymus	1.18E−02	3.5E−04
Thyroid	4.23E−02	2.1E−04
Urinary bladder wall	4.79E−02	1.5E−02
Uterus	1.56E−02	5.7E−04
Parotid gland^a^	4.13E−02	2.1E−02
Submandibular gland^a^	2.74E−02	1.7E−02
Total body	1.18E−02	1.4E−04
Effective dose equivalent (mSv/MBq)	2.21E−02	1.8E−03
Effective dose (mSv/MBq)	2.47E−02	2.8E−03

^a^Absorbed dose calculated using the sphere model

A high variability (variation coefficient greater than 15 %) in measured residence time was found in the urinary bladder contents, brain, stomach, heart and lungs, while high variability in estimated absorbed doses was found only in the urinary bladder wall and stomach wall. This was most likely due to the physical and functional clearance rates within these organs involved with the excretion process.

## Discussion

The aims of this work were to perform PET imaging of a non-human primate and to estimate the human absorbed dose before clinical use of [^18^F]-tetrafluoroborate in human PET studies. Monkeys are genetically closer to humans, so they are supposed to be the closest model of human metabolism. Some PET dosimetry studies with other radiotracers have compared the biodistribution and absorbed radiation dosimetry data obtained from humans and rodents or non-human primates [15, 17, 22, 23]. Despite biodistribution patterns being quite similar between species with all radiotracers, human effective dose using monkey data seems to be overestimated, with exposures to individual organs both over- and underestimated depending on the radiotracer. For example, Doss et al. [23] state that for the hypoxia marker ^18^F-HX4, the effective dose estimated from monkey whole-body imaging is higher (42 mSv/MBq) in comparison with the result obtained from humans (27 mSv/MBq) However, as rodents show excretion patterns significantly different from those of primates, the non-human primate data more accurately estimate the human dosimetry and provide a better approximation. However, it should be noted that despite the biodistribution and metabolism of [^18^F]-tetrafluoroborate which are not expected to be affected by general anaesthesia, it has been reported that different anaesthetic protocols have a significant effect on thyroid and salivary gland ^99m^TcO_4_^−^ uptake in euthyroid cats [24].

In this study, [^18^F]-tetrafluoroborate biodistribution in the cynomolgus macaque closely mirrors the physiologic distribution of NIS. In addition to the thyroid, this radiotracer largely distinguishes other extrathyroidal tissues such as the gastric mucosa, salivary glands (mainly parotids), or olfactory mucosa, where NIS expression has been demonstrated [2, 25]. In more details, [^18^F]-tetrafluoroborate was able to discriminate clearly the thyroid gland with an excellent signal-to-noise ratio even in the first minutes of the study (Fig. 2), as has been demonstrated previously in mice [9]. In the gastric wall, radiotracer uptake was non-uniform, with different areas showing distinct uptake. This fact could be explained because NIS expression in stomach wall is associated—in humans—to mucin-secreting epithelial gastric cells [1, 3], and the distribution of this cell type is restricted to specific areas of the macaque stomach, predominantly in the gastric antrum [26]. Interestingly, epithelial gastric cells that express NIS in human stomach are also more abundant in the antrum [3]; so probably, final [^18^F]-tetrafluoroborate biodistribution in this organ could be similar between macaques and humans. Finally, although [^18^F]-tetrafluoroborate is not able to cross the blood–brain barrier, a slight uptake was observed in the choroid plexus, where NIS expression has also been demonstrated [2]. Interestingly, non-uptake was showed in the testes, despite the fact that NIS transcript and protein are expressed in germinal and Leydig cells of human normal testes [27]. Moreover, [^18^F]-tetrafluoroborate was seen in the organs that are responsible for excretion (kidneys and bladder) or reflect the vascular phase (heart and lungs) with an uptake in the early stage of the study and a rapid clearance. The PET images show no uptake in bones or joints; hence, there is no in vivo release of [^18^F]-fluoride during the time frame studied, as it has been shown in mice studies [9].

Thyroid pathologies (cancer and hyperthyroidism) are commonly diagnosed using iodine in the form of ^131^I, ^123^I, or ^124^I as well with ^99m^Tc-pertechnetate. These iodine substances/radionuclides produce an effective dose (considering a 35 % thyroid uptake), of 24, 0.22 and 15 mSv/MBq, respectively [28], all of which are higher than the dose produced by [^18^F]-tetrafluoroborate estimated in this study (0.025 mSv/MBq), which in turn is higher than that from ^99m^Tc-pertechnetate (0.013 mSv/MBq).

Apart from the thyroid, the stomach is the critical organ for [^18^F]-tetrafluoroborate with an absorbed dose of 0.079 mGy/MBq, followed by the urinary bladder wall. The organs that receive the highest absorbed dose in the ^131^I, ^123^I, or ^124^I studies are also the stomach (0.46, 0.068 and 0.59 mGy/MBq, respectively) and the bladder wall (0.042, 0.06 and 0.52 mGy/MBq, respectively) [29]. On the other hand, doses from ^99m^Tc-pertechnetate to the stomach and bladder are 0.026 and 0.018 mGy/MBq. The relatively high exposure from [^18^F]-tetrafluoroborate to the stomach is likely due to the to NIS expression in the stomach wall.

Absorbed dose to the salivary glands is of interest in patients treated with differentiated thyroid cancer, since salivary dysfunction is the most common side effect associated to therapies with high radioiodine activity. Thus, submandibular and parotid average organ absorbed doses per administered ^131^I activity in differentiated thyroid cancer treatment were estimated in 0.32 and 0.31 mGy/MBq, respectively [21]. These values are one order of magnitude higher than the values reported here for [^18^F]-tetrafluoroborate. In contrast, the absorbed dose to the salivary glands from ^99m^Tc-pertechnetate is 0.0093 mGy/MBq.

In conclusion, [^18^F]-tetrafluoroborate provides quantitative PET images of high quality with an absorbed dose slightly higher than the dose produced by ^99m^Tc-pertechnetate and much lower than the doses from iodine compounds. This fact supports its use for PET imaging of NIS for diagnosis of disease and monitoring treatment for thyroid cancer and hyperthyroidism. However, human studies with [^18^F]-tetrafluoroborate will validate the biodistribution and dosimetry performed in this study with cynomolgus monkeys.

## Conclusions

We have determined the biodistribution and calculated the absorbed doses after intravenous administration of [^18^F]-tetrafluoroborate in cynomolgus monkeys, by whole-body PET imaging and OLINDA calculations methods. The biodistribution closely mirrors physiologic distribution of NIS. The effective dose is 0.025 mSv/MBq, and the critical organs are the stomach wall, the urinary bladder wall, and the thyroid.

## Additional file

Additional file 1:
**3D video of dynamic [18F]-tetrafluoroborate biodistribution.** (MOV 776 kb)

## Abbreviations

NIS: sodium/iodide symporter
TAC: time-activity curve
VOI: volume of interest

## References

[1] Bruno R, Giannasio P, Ronga G, Baudin E, Travagli JP, Russo D (2004). Sodium iodide symporter expression and radioiodine distribution in extrathyroidal tissues. J Endocrinol Invest.

[2] Dohan O, De la Vieja A, Paroder V, Riedel C, Artani M, Reed M (2003). The sodium/iodide symporter (NIS): characterization, regulation, and medical significance. Endocr Rev.

[3] Vayre L, Sabourin JC, Caillou B, Ducreux M, Schlumberger M, Bidart JM (1999). Immunohistochemical analysis of Na symporter distribution in human extra-thyroidal tissues. Eur J Endocrinol.

[4] Micali S, Bulotta S, Puppin C, Territo A, Navarra M, Bianchi G (2014). Sodium iodide symporter (NIS) in extrathyroidal malignancies: focus on breast and urological cancer. BMC Cancer.

[5] Ahn BC (2012). Sodium iodide symporter for nuclear molecular imaging and gene therapy: from bedside to bench and back. Theranostics.

[6] Penheiter AR, Russell SJ, Carlson SK (2012). The sodium iodide symporter (NIS) as an imaging reporter for gene, viral, and cell-based therapies. Curr Gene Ther.

[7] Eschmann SM, Reischl G, Bilger K, Kupferschlager J, Thelen MH, Dohmen BM (2002). Evaluation of dosimetry of radioiodine therapy in benign and malignant thyroid disorders by means of iodine-124 and PET. Eur J Nucl Med Mol Imaging.

[8] Sgouros G, Kolbert KS, Sheikh A, Pentlow KS, Mun EF, Barth A (2004). Patient-specific dosimetry for 131I thyroid cancer therapy using 124I PET and 3-dimensional-internal dosimetry (3D-ID) software. J Nucl Med.

[9] Jauregui Osoro M, Sunassee K, Weeks A, Berry D, Paul R, Cleij M (2010). Synthesis and biological evaluation of [(18)F]tetrafluoroborate: a PET imaging agent for thyroid disease and reporter gene imaging of the sodium/iodide symporter. Eur J Nucl Med Mol Imaging.

[10] Eskandari S, Loo DD, Dai G, Levy O, Wright EM, Carrasco N (1997). Thyroid Na+/I- symporter. Mechanism, stoichiometry, and specificity. J Biol Chem.

[11] ANBAR M, GUTTMANN S, LEWITUS Z (1960). The accumulation of fluoroborate ions in thyroid glands of rats. Endocrinology.

[12] Youn H, Jeong JM, Chung JK (2010). A new PET probe, (18)F-tetrafluoroborate, for the sodium/iodide symporter: possible impacts on nuclear medicine. Eur J Nucl Med Mol Imaging.

[13] Weeks A, Jauregui Osoro M, Cleij M, Blower J, Ballinger J, Blower P (2011). Evaluation of [18 F]-tetrafluoroborate as a potential PET imaging agent for the human sodium/iodide symporter in a new colon carcinoma cell line, HCT116, expressing hNIS. Nucl Med Commun.

[14] Brix G, Zaers J, Adam LE, Bellemann ME, Ostertag H, Trojan H (1997). Performance evaluation of a whole-body PET scanner using the NEMA protocol. National Electrical Manufacturers Association. J Nucl Med.

[15] Brown AK, Fujita M, Fujimura Y, Liow JS, Stabin M, Ryu YH (2007). Radiation dosimetry and biodistribution in monkey and man of 11C-PBR28: a PET radioligand to image inflammation. J Nucl Med.

[16] Stabin MG, Sparks RB, Crowe E (2005). OLINDA/EXM: the second-generation personal computer software for internal dose assessment in nuclear medicine. J Nucl Med.

[17] Anderson CJ, Dehdashti F, Cutler PD, Schwarz SW, Laforest R, Bass LA (2001). 64Cu-TETA-octreotide as a PET imaging agent for patients with neuroendocrine tumors. J Nucl Med.

[18] Antenor-Dorsey JA, Laforest R, Moerlein SM, Videen TO, Perlmutter JS (2008). Radiation dosimetry of N-([11C]methyl)benperidol as determined by whole-body PET imaging of primates. Eur J Nucl Med Mol Imaging.

[19] Wessels BW, Bolch WE, Bouchet LG, Breitz HB, Denardo GL, Meredith RF (2004). Bone marrow dosimetry using blood-based models for radiolabeled antibody therapy: a multiinstitutional comparison. J Nucl Med.

[20] Senthamizhchelvan S, Hobbs RF, Song H, Frey EC, Zhang Z, Armour E (2012). Tumor dosimetry and response for 153Sm-ethylenediamine tetramethylene phosphonic acid therapy of high-risk osteosarcoma. J Nucl Med.

[21] Jentzen W, Hobbs RF, Stahl A, Knust J, Sgouros G, Bockisch A (2010). Pre-therapeutic (124)I PET(/CT) dosimetry confirms low average absorbed doses per administered (131)I activity to the salivary glands in radioiodine therapy of differentiated thyroid cancer. Eur J Nucl Med Mol Imaging.

[22] Doss M, Kolb HC, Zhang JJ, Belanger MJ, Stubbs JB, Stabin MG (2012). Biodistribution and radiation dosimetry of the integrin marker 18F-RGD-K5 determined from whole-body PET/CT in monkeys and humans. J Nucl Med.

[23] Doss M, Zhang JJ, Belanger MJ, Stubbs JB, Hostetler ED, Alpaugh K (2010). Biodistribution and radiation dosimetry of the hypoxia marker 18F-HX4 in monkeys and humans determined by using whole-body PET/CT. Nucl Med Commun.

[24] Schaafsma IA, Pollak YW, Barthez PY (2006). Effect of four sedative and anesthetic protocols on quantitative thyroid scintigraphy in euthyroid cats. Am J Vet Res.

[25] Spitzweg C, Joba W, Eisenmenger W, Heufelder AE (1998). Analysis of human sodium iodide symporter gene expression in extrathyroidal tissues and cloning of its complementary deoxyribonucleic acids from salivary gland, mammary gland, and gastric mucosa. J Clin Endocrinol Metab.

[26] Vidal JD, Mirabile RC, Thomas HC (2008). Evaluation of the cynomolgus monkey stomach: recommendations for standard sampling procedures in nonclinical safety studies. Toxicol Pathol.

[27] Russo D, Scipioni A, Durante C, Ferretti E, Gandini L, Maggisano V (2011). Expression and localization of the sodium/iodide symporter (NIS) in testicular cells. Endocrine.

[28] International Commission on Radiological Protection (ICRP). Radiation dose to patients from radiopharmaceuticals (addendum 2 to ICRP publication 53). Ann ICRP. 1998;28:1–126. 10.1016/s0146-6453(99)00006-8 10840563

[29] International Commission on Radiological Protection (ICRP). Radiation dose to patients from radiopharmaceuticals. ICRP Publication 53. Ann ICRP 1988;18:1–4. 3505163

